# Nectin4 restricts pseudorabies virus infection by blocking gD-Nectin1 interaction

**DOI:** 10.1128/jvi.00788-26

**Published:** 2026-07-20

**Authors:** Yi Wang, Zongyi Bo, Senhong Zhao, Tanveer Ali Fazlani, Fayu Yang, Zhengshan Feng, Tianxing Cao, Zhonghui Zhang, Wenchao Xue, Shuai Chen, Yupan Qiao, Zixiang Zhu, Genhong Cheng, Yiping Wang, Haixue Zheng, Shilei Zhang

**Affiliations:** 1 State Key Laboratory of Animal Disease Control and Prevention, College of Veterinary Medicine, Lanzhou University, Lanzhou Veterinary Research Institute, Chinese Academy of Agricultural Sciences, Lanzhou, China; 2 Joint International Research Laboratory of Agriculture and Agri-Product Safety, Yangzhou University, College of Veterinary Medicine, The Ministry of Education of China, Yangzhou, China; 3 Department of Preventive Veterinary Medicine, Research Center for Swine Diseases, Sichuan Agricultural University, College of Veterinary Medicine, Chengdu, China; 4 Gansu Province Research Center for Basic Disciplines of Pathogen Biology, Lanzhou, China; 5 Department of Microbiology, Immunology and Molecular Genetics, University of California, Los Angeles, California, USA; University of Virginia, Charlottesville, Virginia, USA

**Keywords:** pseudorabies virus, Nectin4, viral entry, virus-host interaction

## Abstract

The alphaherpesvirus *Pseudorabies virus* (PRV), which causes Aujeszky’s disease (AD), primarily infects swine but has zoonotic potential and can infect multiple species, including humans. Upon infection, interferons (IFNs) trigger the expression of hundreds of interferon-stimulated genes (ISGs), thereby establishing a robust cellular antiviral state. However, ISGs exhibiting anti-alphaherpesvirus activity have not yet been fully characterized. Here, we perform an unbiased screen of 268 ISGs and identify Nectin4, also known as PVRL4, as a potent antiviral ISG against alphaherpesvirus, including herpes simplex virus (HSV). Mechanistically, Nectin4 inhibits viral internalization into host cells, in which the IgV domain plays a critical role. Specifically, Nectin4 interacts with the viral gD protein through this domain, thereby inhibiting the interaction between gD and viral receptor, Nectin1, and blocking gD-mediated viral entry. Together, our data identify a previously unrecognized function of Nectin4 in host anti-alphaherpesvirus immunity and highlight a novel cellular barrier that restricts alphaherpesvirus infection.

**IMPORTANCE:**

Interferon-stimulated genes (ISGs) are the major mediators of the host antiviral innate immunity, yet most remain functionally uncharacterized against alphaherpesviruses. Here, we identify interferon-stimulated Nectin4 (PVRL4) as a potent alphaherpesvirus restriction factor. By directly engaging the viral glycoprotein D (gD) and blocking its interaction with the entry receptor Nectin1, Nectin4 suppresses viral internalization. These findings broaden our understanding of the host-alphaherpesvirus interaction and highlight Nectin4 as a potential target for antiviral intervention against alphaherpesviruses.

## INTRODUCTION

Pseudorabies virus (PRV), the causative agent of Aujeszky’s disease (AD), has posed a serious threat to the global swine industry. PRV, a member of the alphaherpesvirinae subfamily, has a double-stranded genomic DNA of approximately 143 kb and contains at least 72 genes encoding 70 different proteins, including envelope glycoproteins, which are crucial for the viral life cycle (1). Among them, glycoprotein C (gC) is essential for initial attachment to heparan sulfate on the cell surface (2, 3), gB and gH/gL are crucial for PRV fusion with the host cell membrane during infection (4, 5), and gD interacts with receptor Nectin1 to promote viral fusion and entry (6, 7). As the primary entry receptor of PRV, Nectin1 is broadly expressed across diverse tissues and cell types and is especially abundant in the brain, which in turn renders a wide variety of cells vulnerable to PRV infection. Although PRV primarily infects swine, it can also infect a broad range of mammals, including cattle, sheep, and canines (8). In recent years, accumulating evidence suggests that PRV has become a zoonotic pathogen that can infect humans through infected pigs or pork (9–11). More importantly, the variant PRV strains, which are more virulent than classical strains, have appeared and spread in China since 2011 (12, 13). To date, no commercial vaccine or approved antiviral drugs are able to control the spread of diseases effectively. Therefore, investigation of viral replication and virus-host interactions is essential for identifying new cellular targets for PRV control.

Antiviral innate immune response is the first line of defense against viral infection. Interferons (IFNs), the primary executors of antiviral innate immunity, induce the expression of hundreds of IFN-stimulated genes (ISGs), which target different phases of the viral life cycle (14). Numerous studies have demonstrated that ISGs exert antiviral effects on PRV either by directly interfering with the PRV life cycle (15, 16) or by indirectly modulating cellular processes such as apoptosis, autophagy, or the DNA damage response, thereby limiting PRV replication and spread. Cholesterol 25-hydroxylase (CH25H) and the IFN-induced transmembrane protein (IFITMs) family can dampen viral entry into cells (17), while the porcine Mx protein (Mx1 and Mx2) restricts the synthesis of PRV early genes (18). Moreover, ISG15 (16), ISG20 (19), and DDX56 (20) impose their antiviral activities against PRV by facilitating interferon signaling. Although the induction of these ISGs establishes a strong host antiviral state against PRV, it is not sufficient to account for the anti-PRV effect of interferons, suggesting that some ISGs with anti-PRV activity as IFN effectors have not been fully characterized.

In this study, we performed a high-throughput screen using an ISG library comprising 268 different ISGs to determine genes that robustly inhibit PRV infection in cells. We successfully identified a panel of ISGs with restriction activity against PRV replication, including IFITMs, PARP11, KMO, and TRIM5. Among them, Nectin4 emerged as the most potent inhibitor of both PRV and HSV-1 replication. We found that Nectin4 suppressed PRV infection at the entry stage through its IgV domain-mediated interaction with viral gD protein, thereby interfering with the gD-Nectin1 interaction, which is critical for PRV entry. This study highlights that the interferon-stimulated Nectin4 plays a key role in the cellular defenses against alphaherpesvirus.

## RESULTS

### ISG expression screen identifies restriction factors against pseudorabies virus

**Fig 1:**
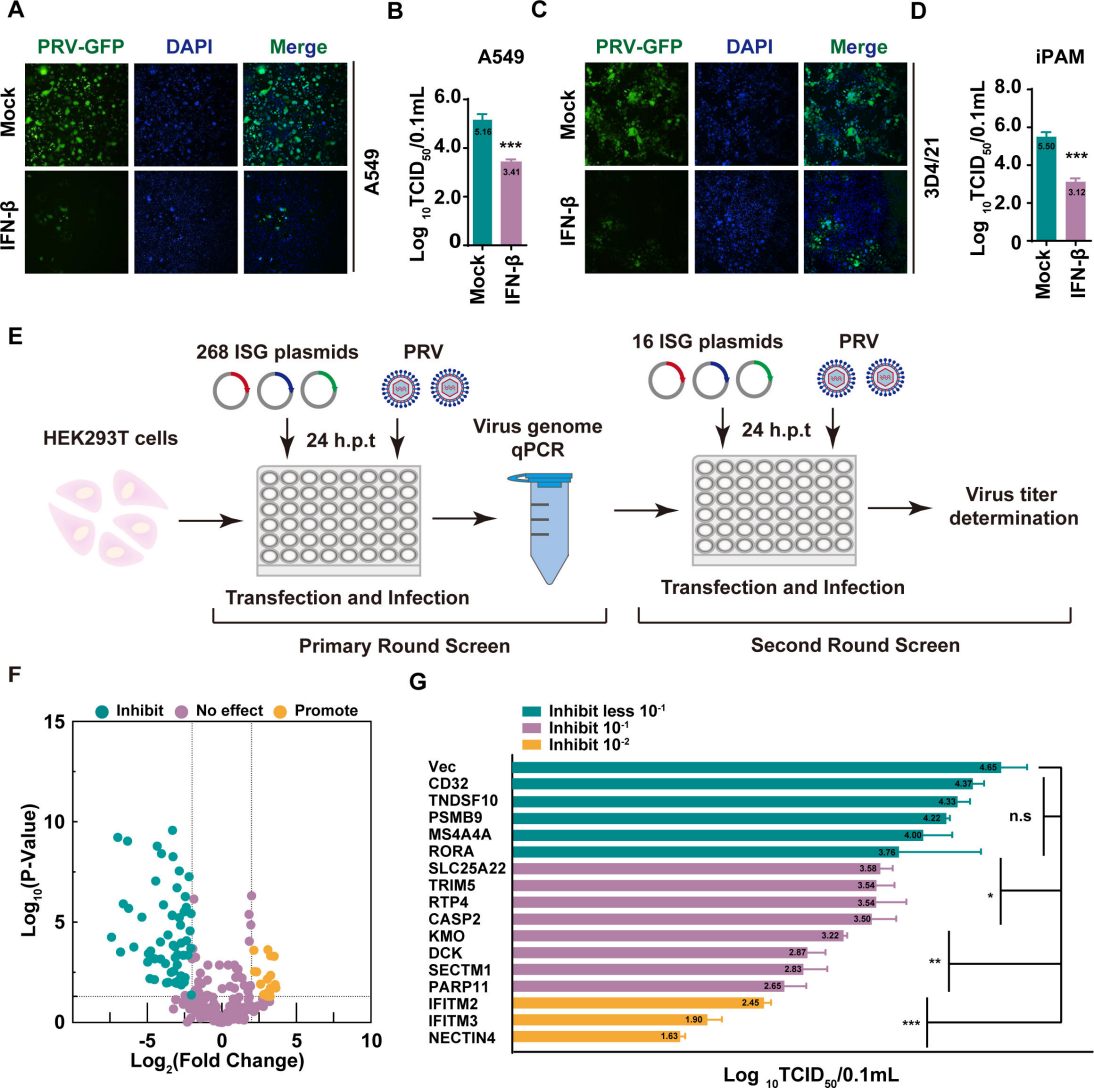
ISG screen identifies restriction factors for Pseudorabies virus. (**A and B**) A549 cells and (**C and D**) iPAM cells were treated with human or porcine IFN-β for 12 h and infected with PRV-GFP (MOI of 0.1) for 24 h. GFP fluorescence was observed (**A and C**), and viral titers were measured (**B and D**). (**E**) Schematic of ISG library screening. Two rounds of screening were performed to enrich the gene of interest. HEK293T cells were transfected with 268 ISG eukaryotic expression plasmids from the ISG library and infected with PRV (MOI of 0.1) for 24 h. The cell culture medium was collected to detect the viral genome copy number by RT-qPCR (**F**). Then, HEK 293T cells were transfected with 16 high-scoring targets for 24 h and infected with PRV (MOI of 0.1) for 24 h. (**G**) Viral titer determination was performed by viral TCID_50_ assay. An empty vector was used as a negative control. *P < 0.05, **P < 0.01, and ***P < 0.001 by unpaired *t*-test. Graphs show mean ± SD.

Interferons show broad-spectrum antiviral activity by triggering the expression of ISGs. First, we demonstrated that Type-I interferon can significantly inhibit PRV replication. We used human or porcine IFN-β to treat A549 cells or the immortalized porcine alveolar macrophage cell line (iPAM 3D4/31), followed by PRV-GFP (multiplicity of infection [MOI] of 0.1) challenge. As expected, Type-I interferon exhibited potent antiviral activity against PRV (Fig. 1A through D). To single out the key ISGs in IFN-mediated antiviral effects, we performed a two-round screening using an ISG library comprising 268 different ISGs (Fig. 1E). In the first round, we individually transfected 268 human ISGs into HEK293T cells and then challenged cells with PRV (21). At 24 h post-infection, the cell supernatant was collected for viral genome copy quantification by qPCR. As shown in Fig. 1F, numerous ISGs showed distinct antiviral phenotypes (73/268, -Log2FC > 2). Then, we selected 16 genes with the most significant antiviral activity for secondary screening, and 11 out of 16 genes inhibited PRV infection, resulting in at least a 10-fold reduction in viral titers compared with the negative control (Fig. 1G). Interestingly, Nectin4, which has been reported as the receptor of the measles virus, was found to be the most effective restriction factor against PRV infection (Fig. 1G). Transcription of Nectin4 could be activated by the stimulation of interferon, PolyI: C, and PolydA:dT (Fig. S1A and B). Consistently, Type I interferons increased the expression of Nectin4 protein in macrophages (Fig. S1C and D), suggesting that Nectin4 is an ISG with potent antiviral activity against PRV. To rule out possible cytotoxicity caused by the overexpression of foreign genes, we examined the cell viability after the expression of ISGs by using the CCK-8 assay. Most ISGs with potent antiviral activity against PRV, including Nectin4, did not show significant cytotoxicity (Fig. S1F).

### Nectin4 inhibits alphaherpesvirus replication

**Fig 2:**
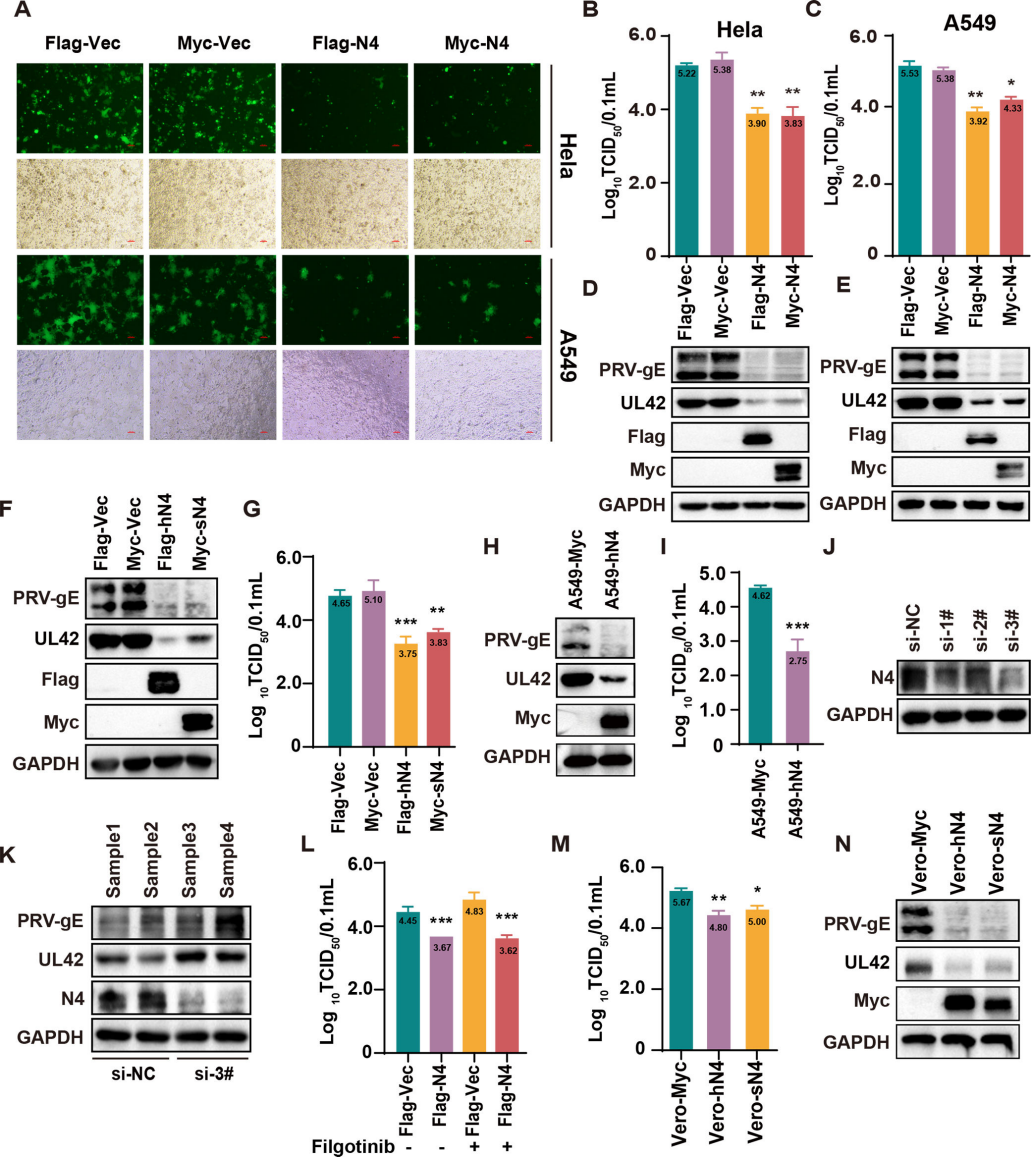
Nectin4 is a potent restriction factor against herpesvirus. (**A–E**) HeLa cells and A549 cells were transfected with the Nectin4 plasmid tagged with Flag or Myc for 24 h and then infected with PRV (MOI of 0.1) for 24 h. (**A**) The expression of GFP in the cells infected with PRV. (**B and C**) Viral replication was detected by the TCID_50_ assay. (**D and E**) Expression of viral proteins gE and UL42 was detected by Western blot. The control vector with the same backbone was used as a negative control. To compare the antiviral activity of Nectin4 from different species, HeLa cells were transfected with human or porcine Nectin4, followed by PRV infection (MOI of 0.1) for 24 h. PRV-gE and UL42 expressions were detected by Western blot (**F**), and the viral titer was determined by TCID_50_ assay (**G**). A549 stable cells expressing Nectin4 were infected with PRV (MOI of 0.1) for 24 h. PRV-gE and UL42 expressions were detected by Western blot (**H**), and viral replication was quantified by TCID_50_ assay (**I**). Small interfering RNA (siRNA)-mediated Nectin4 knockdown in Caov3 cells was performed, and the knockdown efficiency was detected by Western blot (**J**). Western blot detection of viral gE and UL42 in siRNA3#-transfected Caov3 cells (**K**). After transfection with the indicated plasmids, HeLa cells were treated with JAK1 inhibitor filgotinib (Fil) for 6 h and then infected with PRV (MOI of 1) with filgotinib for an additional 24 h. Viral replication was detected by the TCID_50_ assay (**L**). PRV replication in Nectin4 transiently expressed Vero cells was detected by TCID_50_ assay (**M**) and Western blot (**N**). *P < 0.05, **P < 0.01, and ***P < 0.001 by unpaired *t*-test. Graphs show mean ± SD.

Nectin4 is highly expressed in ovarian cancer cell Caov3 but is expressed at very low levels in many other cell types, such as A549, HeLa, Huh7, and HEK293T cells (Fig. S1E). To further determine the inhibitory effect of Nectin4 on alphaherpesviruses, HeLa cells and A549 cells were transfected with Myc-tagged or Flag-tagged Nectin4 and then challenged with PRV-GFP. Fluorescent imaging indicated that Nectin4 overexpression suppressed PRV-GFP replication in both HeLa and A549 cells (Fig. 2A). Consistent with these observations, RT-qPCR demonstrated that transcription of PRV-gB was notably declined (Fig. S2A and B), and the TCID_50_ assay measuring infectious titer at 24 hpi showed a marked decrease in Nectin4-overexpressing cells (Fig. 2B and C). Western blotting (WB) results further revealed a significant reduction in PRV-gE and UL42 protein levels in Nectin4-overexpressing cells (Fig. 2D and E). Furthermore, Nectin4 also inhibited HSV-1 replication, as demonstrated by Western blotting and RT-qPCR (Fig. S2C and D).

Since pig is the only natural host of PRV, we investigated whether porcine Nectin4 also exhibits similar antiviral activity. HEK293T cells were transiently transfected with either human or porcine Nectin4 and then challenged with PRV-GFP for 24 h. The results revealed that both possessed a comparable antiviral activity (Fig. 2F and G; Fig. S2E and F). Furthermore, A549 stably expressing Nectin4 was constructed to validate the antiviral activity, and similar results were obtained (Fig. 2H and I). Next, we introduced small interfering RNA (siRNA) specific for Nectin4 into Caov3 cells and determined the knockdown efficacy by western blot assay (Fig. 2J). Contrary to the overexpression results, PRV replication was increased in Nectin4 knockdown cells (Fig. 2K). Together, Nectin4 exhibits a potent inhibitory effect in PRV infection.

### Anti-PRV activity of Nectin4 is independent of interferon signaling

Given that some ISGs are feedback-induced to regulate interferon signaling, we thus asked whether Nectin4 exerts antiviral activity through amplifying the interferon signaling. To this end, we used the JAK1 inhibitor filgotinib (Fil) to inhibit the interferon signaling and further tested the antiviral activity of Nectin4. First, the blocking activity of Fil on interferon signaling was determined. Poly(dA:dT), a synthetic analog of B-DNA, could induce ISG20 expression in both HeLa and A549 cells but failed in the context of Fil (Fig. S2I and J). Moreover, the activity of Fil was not attributable to cytotoxicity as no significant cytotoxicity was observed by measuring cell viability with the CCK-8 assay (Fig. S2G and H). HeLa cells were transfected with Flag-tagged Nectin4, then alone or pretreated with Fil for 6 h, followed by PRV infection for an additional 24 h. As shown in Fig. 2L, even though interferon signaling was inhibited by Fil, the antiviral effect of Nectin4 is not weakened. To further confirm this, Vero cells, which cannot naturally produce type I interferons, were genetically engineered to express Nectin4 consistently. Consistent with the above results, Nectin4 still possesses significant antiviral activity against PRV (Fig. 2M and N). These results indicated that the inhibitory effect of Nectin4 on PRV infection is independent of interferon signaling.

### Nectin4 interferes with alphaherpesvirus entry into the cells

**Fig 3:**
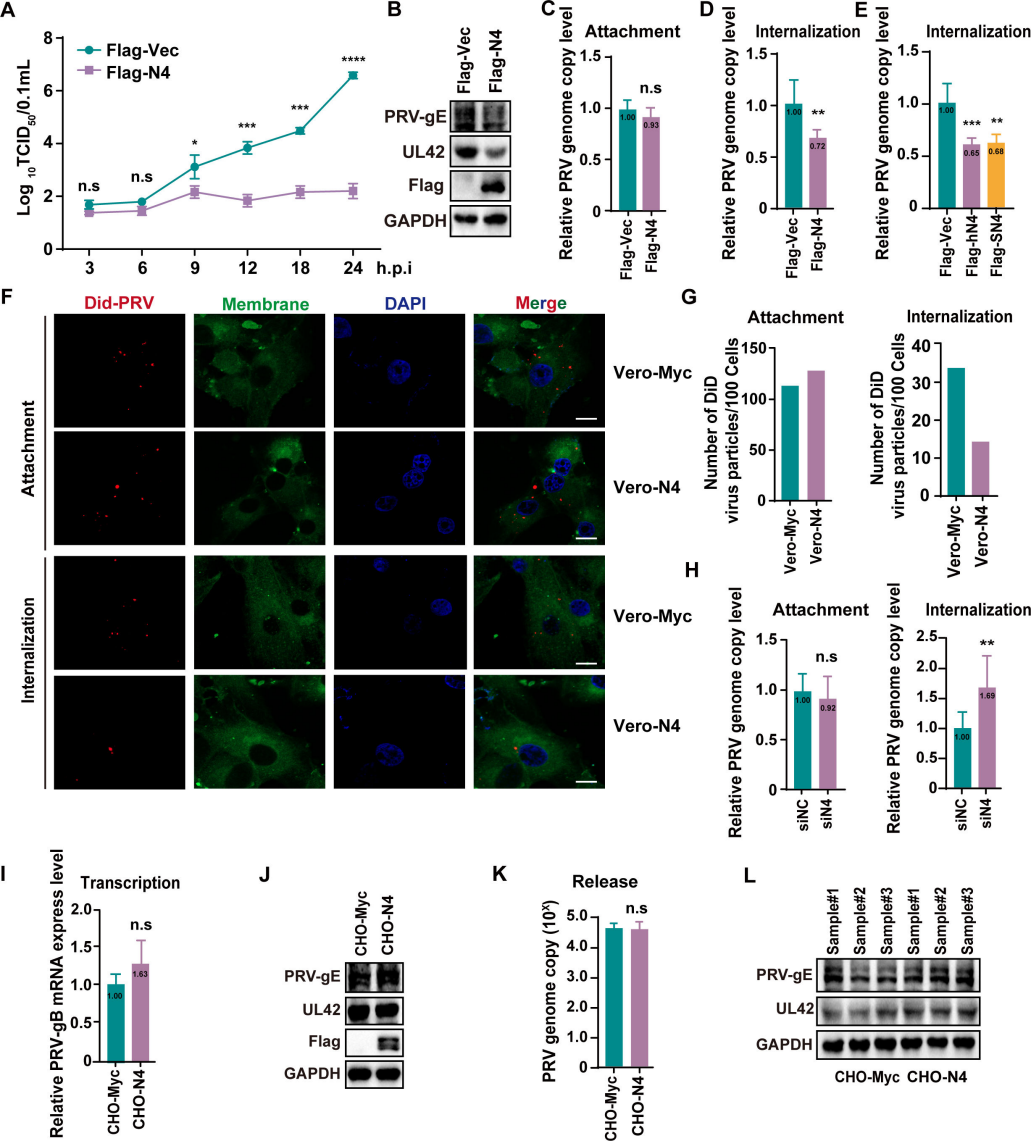
Nectin4 targets the internalization of the PRV life cycle. HEK293T cells were infected with PRV (MOI of 1) for 24 h after transfection with Nectin4 and were collected at different times for TCID_50_ assay (**A**). PRV-gE and UL42 protein expression was detected after infection for 6 h by Western blot (**B**). HeLa cells transfected with the Nectin4 plasmid were infected with PRV (MOI of 1) for attachment (**C**) and internalization assays (**D**). PRV was incubated with the cells on ice for 1 h, and viral genome copy number was determined by qPCR after three washes using cold phosphate-buffered saline (PBS). Following attachment on ice, PRV internalized into HeLa cells for 1 h at 37℃. After rinsing with acidic buffer to remove the noninternalized viral particles, the viral genome was assessed by qPCR. Nectin4 from different species was transfected into HeLa cells for the PRV internalization assay (**E**). Virions were visualized by labeling with the lipophilic dye Did. Vero cells stably expressing Nectin4 were incubated with Did-PRV (MOI of 1) at 4℃, followed by or not by incubation at 37℃ for 1 h. The bound or internalized virions were assessed by confocal microscopy (**F**) (Did-PRV, red; cell membrane, green; cell nucleus, blue). The ratio of virus particles (Did, red) to cells (DAPI, blue) was analyzed using a high-content imaging system. The data are presented as the average number of red virus particles tracked within every 100 cells (**G**). Each group of scans covers at least 5,000 cells. Caov3 cells were transfected with siRNA against Nectin4 for 36 h, then infected with PRV (MOI of 1) for attachment and internalization assays, and the viral genome was assessed by qPCR (**H**). Scramble siRNA was used as a negative control. The PRV genome was transfected into CHO cells stably expressing Nectin4 for 24 h. The intracellular PRV-gB transcription level (**I**), viral protein expression (**J**), and viral genome copy number in the supernatant were determined (**K**). (**L**) The virus released from the supernatant was infected with Vero cells for 24 h, and virus replication was determined by Western blot. *P < 0.05, **P < 0.01, and ***P < 0.001 by unpaired *t*-test. Graphs show mean ± SD.

Since Nectin4 restricts PRV replication without augmenting IFN responses, we next investigated which stage of the viral replication cycle was targeted by Nectin4. First, we quantified the viral replication at 3, 6, 9, 12, 18, and 24 h post-infection by TCID_50_ assay and demonstrated that progeny viral particles in control cells were more than those in Nectin4-overexpressing cells at 9 h post-infection (Fig. 3A). The viral protein levels were also determined to be reduced at 6 h post-infection (Fig. 3B). These data suggested that Nectin4 impacted an early step in the viral replication cycle. Given that Nectin4 is a cell surface protein, we hypothesized that Nectin4 may target PRV attachment or internalization. We performed a surface binding assay with PRV on the Flag vector- and Flag-tagged Nectin4-transfected HeLa cells at 4°. No statistically significant difference in bound viral genomes was detected by qPCR (Fig. 3C). The gC of PRV binds to heparin to mediate virus attachment. HeLa cells with heparinase significantly decreased PRV attachment, while Nectin4 overexpression still did not affect viral attachment (Fig. S2K), suggesting that Nectin4 does not affect the surface binding of PRV. HeLa cells were incubated with PRV for 1 h at 4℃, followed by 1 h incubation at 37°C and subsequent removal of noninternalized viral particles by extensive acid washes. Viral internalization was quantified by qPCR and revealed that Nectin4 overexpression significantly inhibited PRV internalization (Fig. 3D). Nectin4 from different species showed similar inhibitory activity on PRV internalization (Fig. 3E). The stage of the HSV-1 replication cycle targeted by Nectin4 was also determined. As shown in Fig. S2L and M, Nectin4 impaired HSV-1 internalization, but not attachment, consistent with its effect on the PRV life cycle. To further confirm this, purified PRV particles labeled with the red lipophilic membrane dye Did were used to visualize viral binding and internalization. No significant difference was observed in viral particle binding to Nectin4-stably expressing Vero cells, whereas viral entry was markedly reduced (Fig. 3F and G). In addition, we examined the role of endogenous Nectin4 in PRV internalization in Caov3 cells using specific siRNA. Specific knockdown of Nectin4 increased viral internalization without affecting viral attachment (Fig. 3H).

Chinese hamster ovary (CHO) cells, which lack the PRV entry receptor Nectin1, serve as an ideal cell model for investigating post-entry stages of the PRV life cycle. To exclude the possibility that Nectin4 affects the stages of the life cycle following internalization, we transfected the PRV genome into the CHO cells. The transcription and translation of the viral genome were evaluated by qPCR and WB assay, and no significant differences were observed in wild-type and Nectin4-stably expressing CHO cells (Fig. 3I and J). The viral genome copies in the supernatant were quantified by qPCR to determine the production of the progeny virus particles (Fig. 3K), which were demonstrated to be comparable in these cell lines. In addition, we collected the progeny viruses released by the wild-type or Nectin4-stably expressing CHO cells. The progeny virus was infected with Vero cells for 24 h. Consistent with this result, the viral protein expression in Vero cells infected with the above supernatants showed no difference (Fig. 3L). Taken together, these results reveal that Nectin4 interferes with PRV internalization during the entry stages.

### The IgV-like domain of Nectin4 is required for its antiviral activity

**Fig 4:**
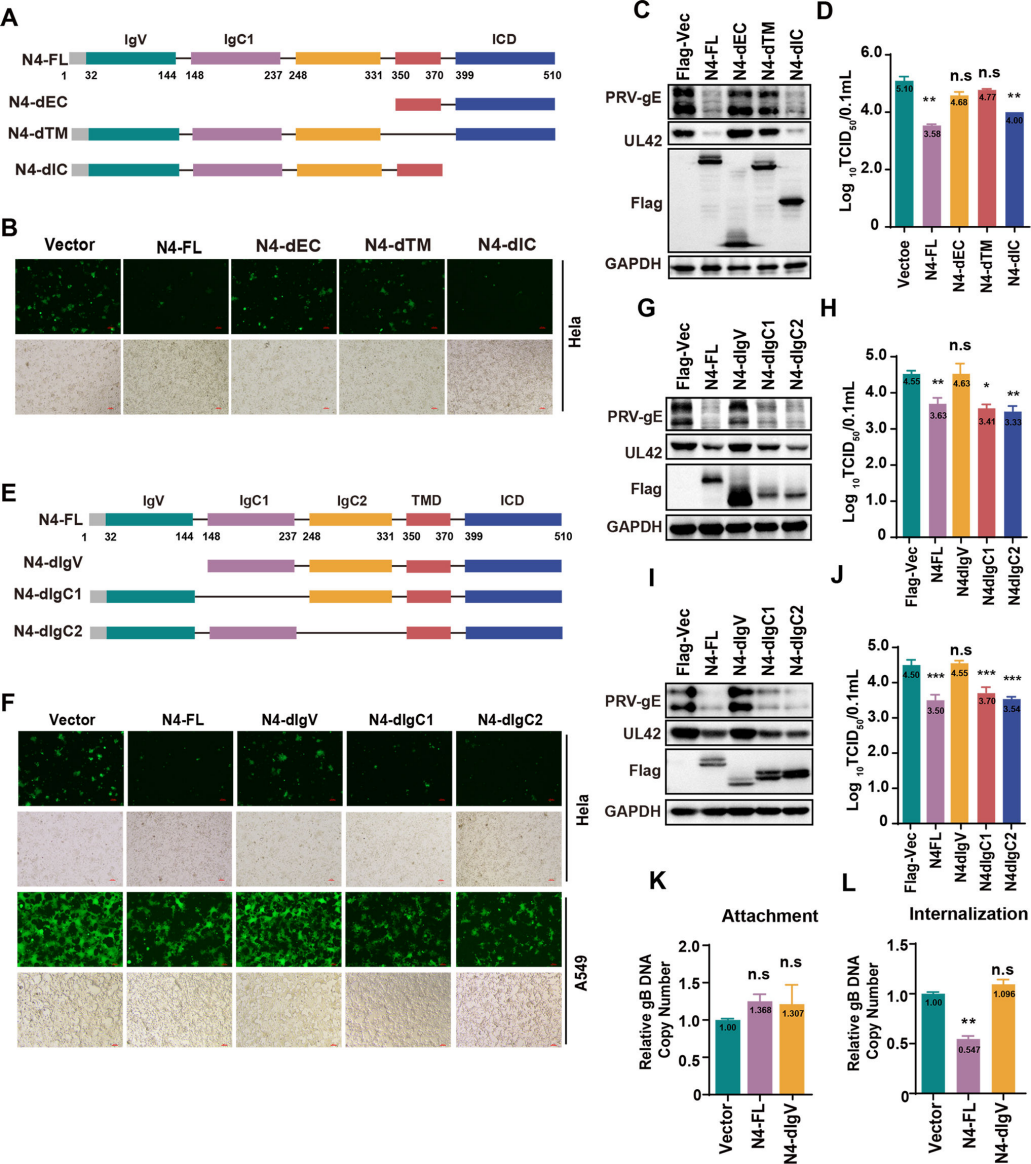
The extracellular IgV-like domain is critical for the antiviral activity of Nectin4. (**A**) Truncated mutants of Nectin4 were constructed by deleting different functional domains. N4-FL: full-length Nectin4; N4-dEC: mutant with deletion of the extracellular segment; N4-dTM: mutant with deletion of the transmembrane domain; N4-dIC: mutant with deletion of the intracellular segment. Mutants were transfected into HeLa cells for 24 h and then infected with PRV-GFP (MOI of 0.1) for 24 h. PRV replication was determined by GFP fluorescence imaging (**B**), Western blot detection of PRV-gE and UL42 (**C**), and TCID_50_ assay for viral titration (**D**). (**E**) The IgV-like, IgC1-like, and IgC2-like domain alone deletion mutant was constructed and transfected into HeLa and A549 cells for 24 h and then infected with PRV-GFP (MOI of 0.1) for 24 h. PRV replication was determined by GFP fluorescence imaging (**F**), Western blot detection of PRV-gE and UL42 (**G** for HeLa and **I** for A549), and TCID_50_ assay for viral titration (**H** for HeLa and **J** for A549). HeLa cells were transfected with IgV-like deletion mutants for 24 h and then infected with HSV-1 (MOI of 1) for 24 h. HeLa cells were transfected with IgV-like deletion mutants for 24 h and then infected with PRV (MOI of 1) for attachment (**K**) and internalization assays (**L**). *P < 0.05, **P < 0.01, and ***P < 0.001 by unpaired *t*-test. Graphs show mean ± SD.

As a type I transmembrane glycoprotein, Nectin4 contains an extracellular (EC) domain, a transmembrane (TM) domain, and an intracellular (IC) domain. To further elucidate the antiviral mechanism of Nectin4, we constructed a set of Nectin4 extracellular, transmembrane, and intracellular domain deletion mutants and investigated which domain is required for antiviral activity (Fig. 4A). Fluorescent imaging indicated that deletion of either the EC or TM domain abolished the ability of Nectin4 to suppress PRV replication, whereas deletion of the IC domain retained antiviral activity (Fig. 4B). Consistently, analyses of viral protein expression and progeny virus titer further demonstrated that the EC and TM domains of Nectin4 are critical for its antiviral activity (Fig. 4C and D). Given that Nectin4 acts at the internalization stage of the PRV life cycle, we hypothesized that the TM domain is critical for Nectin4 localization on the cell surface, where the Nectin4 EC domain interacts with the viral surface protein, thereby blocking viral internalization. Similar to its family members, Nectin4 has one IgV-like domain (IgV) and two IgC-like domains (IgC1 and IgC2) in its extracellular domain. To characterize the minimal regions of the EC domain, we designed a series of Nectin4 EC deletion constructs (Fig. 4E). Deletion of the IgV domain completely abolished the antiviral activity of Nectin4 in HeLa (Fig. 4F, G and H), A549 (Fig. 4F, I and J), and HEK293T cells (Fig. S3A and B), whereas deletion of either IgC1 or IgC2 domains did not affect antiviral activity. Next, we verified the antiviral effect of the IgV domain against HSV-1 and found that deletion of the IgV domain failed to inhibit HSV-1 replication (Fig. S3C and D). Finally, deletion of the IgV domain also eliminated the ability to inhibit viral internalization (Fig. 4K and L), consistent with the above findings that Nectin4 suppresses viral entry. Collectively, the IgV domain of Nectin4 is required for its antiviral activity.

### Nectin4 interacts with PRV gD and gK

**Fig 5:**
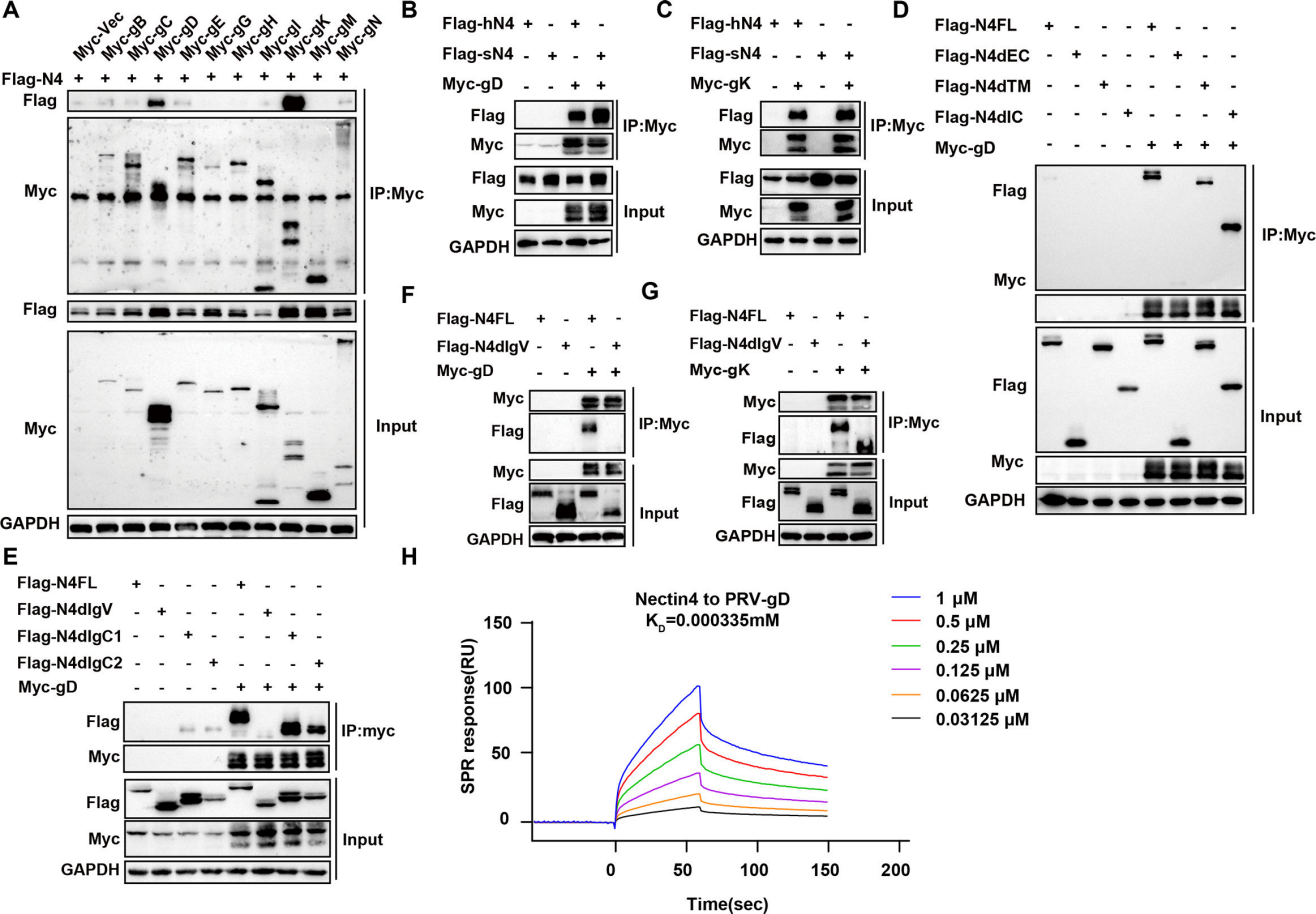
Nectin4 interacts with PRV gD and gK. (**A**) HEK 293T cells were transfected with Flag-tagged human Nectin4 and either Myc-empty vector or Myc-tagged PRV glycoprotein expression plasmids. Cells were lysed at 4℃ and subsequently incubated with Myc-magnetic beads at 4℃ for 2 h. The interaction of Nectin4 with PRV glycoproteins was tested by Western blot. Flag-tagged human or porcine Nectin4 was introduced into HEK293T cells with either Myc-tagged PRV-gD or PRV-gK. The interaction of different species Nectin4 with PRV-gD (**B**) or PRV-gK (**C**) was determined by Western blot. (**D and E**) Myc-tagged gD was cotransfected with Flag-tagged human full-length Nectin4 or different domain deletion plasmids in the HEK293T cells. Immunoprecipitation assays were performed to determine the domain on Nectin4 that is essential for its interaction with gD. The full-length Nectin4 and its IgV-deletion mutant were transfected with Myc-gD (**F**) or Myc-gK (**G**) to evaluate the role of the IgV domain in their interaction. (**H**) Surface plasmon resonance (SPR) characterization of Nectin4 interaction with PRV gD. Representative SPR data for various concentrations of recombinant human Nectin4 protein binding to the recombinant PRV gD protein.

The viral surface proteins are key determinants in the infection, allowing the virus to enter host cells by binding to cellular receptors. Thus, we postulated that Nectin4 might interact with envelope glycoproteins of PRV. To address this, we screened the potential interaction of Nectin4 with each glycoprotein and found that gD and gK interacted with Nectin4 in HEK293T cells (Fig. 5A). These interactions between Nectin4 and viral proteins were conserved across species, as demonstrated in Fig. 5B and C that Nectin4 derived from either human or pig can interact with gD and gK. We further performed co-immunoprecipitations (Co-IPs) with truncated Nectin4 proteins, as well as the full-length protein (Fig. 5D and E). The results demonstrated that it is the EC domain, in particular the IgV domain, that interacts with gD, which is consistent with our finding that IgV is critical for the antiviral activity of Nectin4. We next investigated whether IgV is also essential for interaction with gK. In contrast to gD, gK interacted with both full-length Nectin4 and IgV-deletion mutant, indicating the IgV domain is not the dominant determinant for the interaction with gK (Fig. 5F and G). Further surface plasmon resonance (SPR) assays were performed to assess the binding capacity between Nectin4 and gD. The analysis of the kinetic plots revealed a dissociating constant (KD) of 335 nM, demonstrating a moderate affinity between Nectin4 and gD (Fig. 5H).

### Nectin4 disrupts the interaction of gD and Nectin1

Given that Nectin4 interacts with gD and gK, we further investigate the role of the interaction in Nectin4-mediated viral entry blocking. First, we generated a recombinant PRV by replacing UL53 (the gene encoding gK) with GFP, which cannot express the gK protein. The growth kinetics of PRV-ΔgK were measured to determine whether there was any difference in the *in vitro* replication of the recombinant virus compared with PRV-WT. Viral replication in both intracellular segments and supernatant was quantified, revealing that the PRV-WT replication increased over the course of infection, whereas no PRV-ΔgK replication was observed in the supernatant (Fig. S4A). These results suggested that gK is not essential for PRV intracellular replication but is critical for viral release, which is consistent with the findings of previous publications (22). Moreover, the antiviral activity of Nectin4 against PRV-WT and PRV-ΔgK was compared. Viral replication was assessed by fluorescence imaging (Fig. S4B), qPCR (Fig. S4C), and Western blotting (Fig. S4D), showing that both WT and gK-deleted PRV were suppressed by Nectin4, but not by IgV-deleted Nectin4. These results indicate that Nectin4 inhibits PRV entry through a mechanism independent of gK.

**Fig 6:**
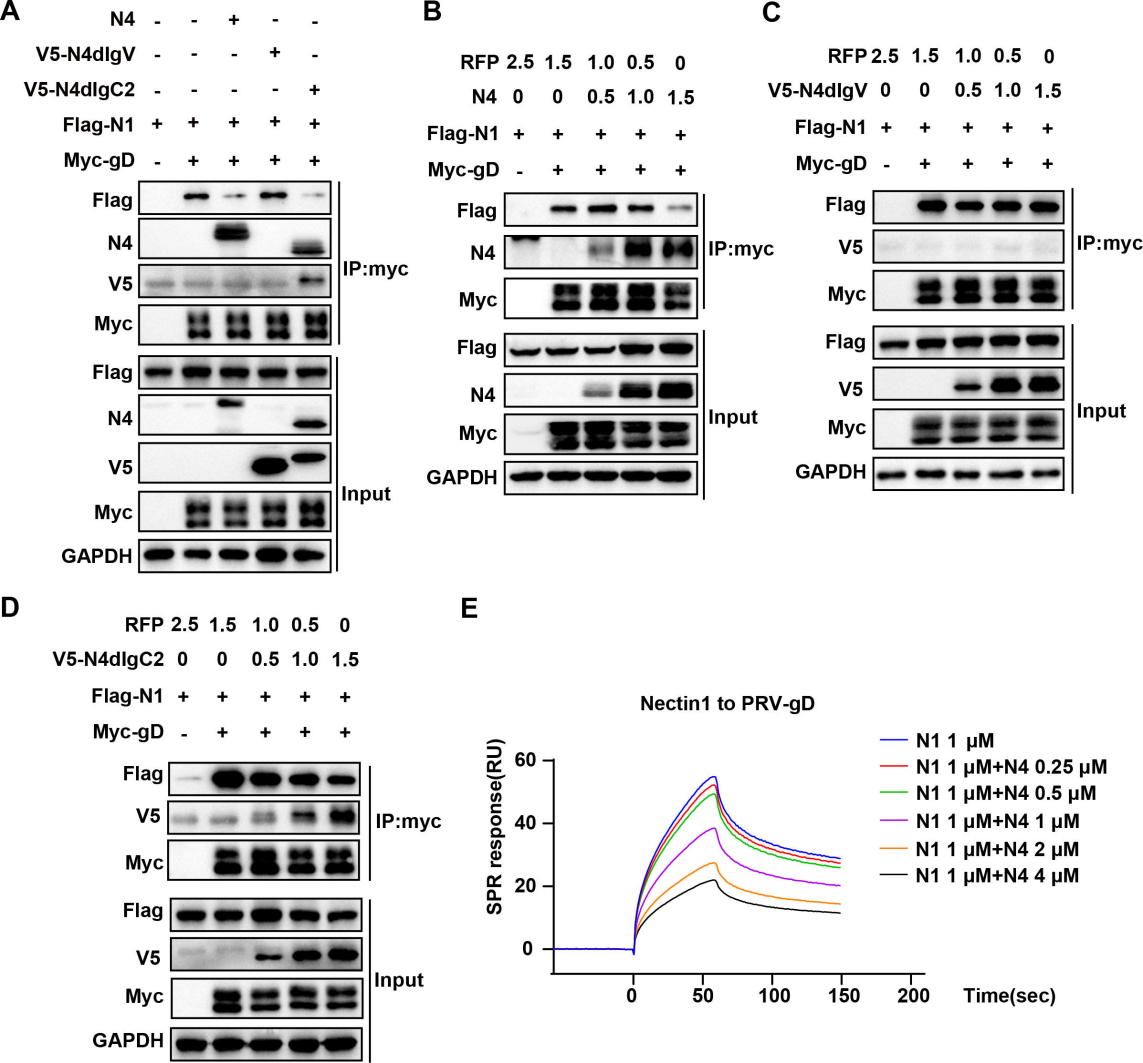
Nectin4 competitively attenuates gD and Nectin1 interaction. (**A**) HEK293T cells were cotransfected with Flag-tagged Nectin1, Myc-empty vector or Myc-PRV-gD, and either full-length or mutant Nectin4 plasmid. Protein samples were lysed at 4°C and incubated with Myc-magnetic beads for 2 h. The Nectin1-PRV-gD interaction was assessed by Western blot. The interaction between Nectin1 and gD was evaluated under different doses of Nectin4 and its mutants. HEK293T cells were cotransfected with Flag-tagged Nectin1, Myc-tagged PRV-gD, and increasing amounts of full-length Nectin4 (**B**), V5-tagged IgV-deletion (**C**), or V5-tagged IgC2 deletion mutants (**D**). The RFP control vector was included as a negative control. Cell lysates were incubated with Myc-magnetic beads at 4°C for 2 h, and the Nectin1-gD interaction was analyzed by Western blot. (**E**) The Nectin1-gD interaction under increasing doses of Nectin4 was determined by SPR. The Nectin4 protein was injected at increasing concentrations onto a surface precoated with a saturated mixture of Nectin1 and PRV-gD proteins. The binding affinity between PRV-gD and Nectin1 was measured by monitoring changes in resonance units (RU) in response to Nectin4 binding.

Given that the interaction with gD is critical for Nectin1-mediated PRV entry and that the Nectin family has a similar extracellular structure, we asked whether Nectin4 affects Nectin1 binding to gD. To address this, we evaluated the interaction between Nectin1 and gD in the presence of Nectin4 and its truncations. Nectin4 and its mutant lacking the IgC2 domain markedly suppressed the interaction between Nectin1 and gD, whereas Nectin4 with IgV deletion failed to block this interaction (Fig. 6A). To confirm this, dose-dependent inhibition was further assessed. As shown in Fig. 6B and D, Nectin4 and its IgC2 mutant exhibited a dose-dependent inhibitory activity on the Nectin1-gD interaction. However, the Nectin1-gD interaction was stable with the increasing amounts of Nectin4-IgV mutants (Fig. 6C). These results provided evidence that Nectin4 inhibits gD binding to Nectin1 through the IgV domain, but not the IgC2 domain, which is consistent with the antiviral domain in Fig. 4. Further SPR experiments were performed to evaluate the blocking effect of Nectin4 on the binding ability of gD and Nectin1. The addition of Nectin4 inhibited the interaction between gD and Nectin1 *in vitro* (Fig. 6E). Together, Nectin4 disrupted the interaction of gD and Nectin-1, thereby inhibiting PRV infection.

## DISCUSSION

**Fig 7:**
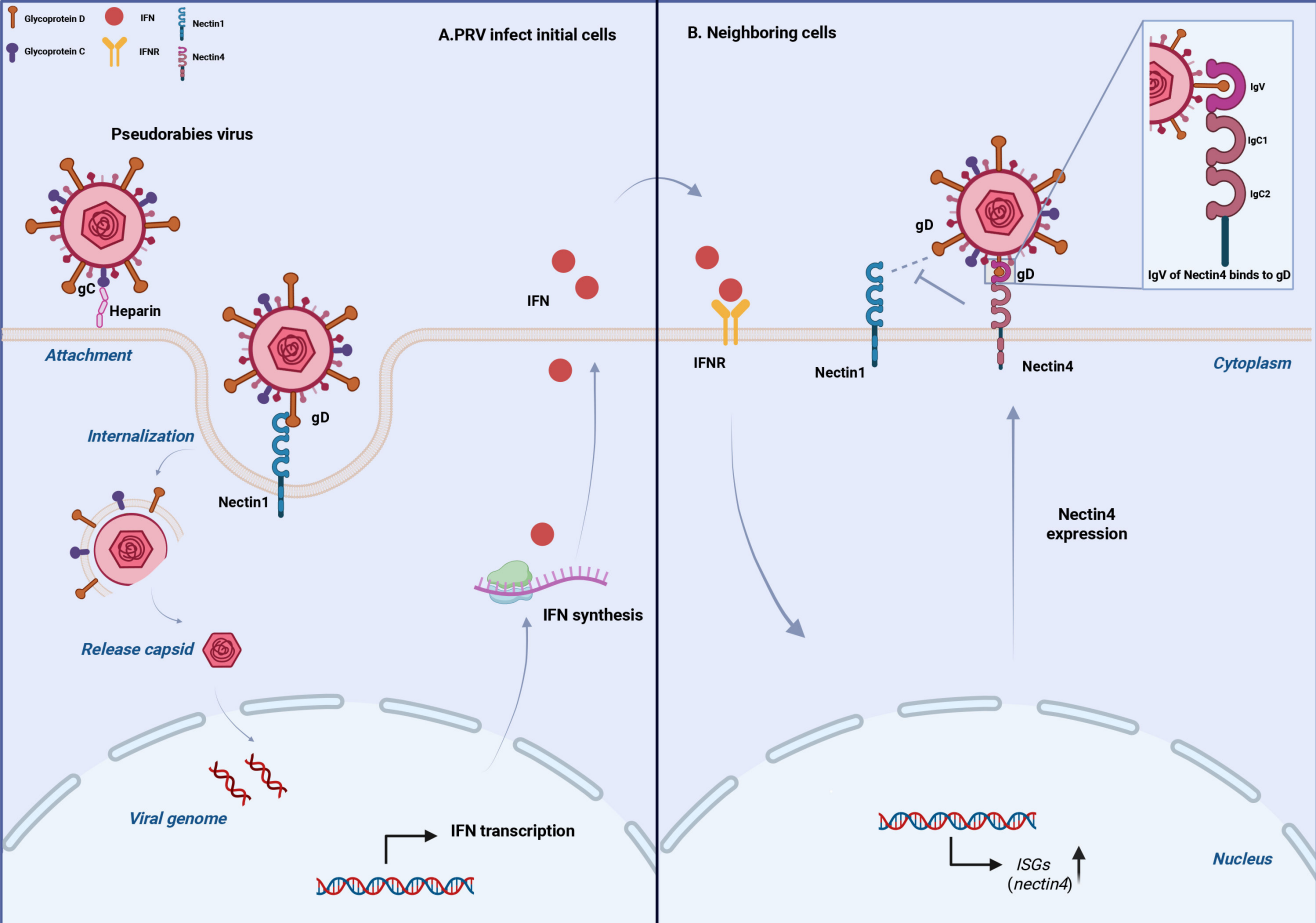
Schematic illustrating the mechanism by which Nectin4 restricts pseudorabies virus infection through blocking gD-Nectin1 interaction. PRV infection involves multiple steps, including attachment, internalization, membrane fusion, and genome release. Initial attachment is primarily mediated through interactions of viral glycoprotein gC with cell-surface heparan sulfate proteoglycans. Receptor binding by gD initiates the fusion cascade and viral internalization. Upon PRV infection, the interferon signaling pathway is activated (left), subsequently upregulating the expression of interferon-stimulated gene Nectin4 in neighboring cells. Nectin4 is located on the cell membrane and binds to gD through its IgV domain, preventing viral internalization (right). Figure created with BioRender (biorender.com).

Despite extensive studies on the antiviral activities of interferons against various viruses, limited knowledge exists regarding interferon-mediated host defense against PRV. In this study, we performed an interferon-stimulated gene library-based screen to identify the potent host restriction factors against PRV. We identified interferon-stimulated Nectin4 as a powerful restriction factor for alphaherpesviruses, including PRV and HSV-1, and subsequently demonstrated that it functions at the entry stage of the viral life cycle via its IgV domain. Mechanistically, we found that Nectin4 binds to PRV gD protein, thereby impairing the interaction between gD and the PRV entry receptor Nectin1 and preventing PRV internalization into host cells (Fig. 7). These findings demonstrate the critical role of Nectin4 in interferon-mediated antiviral innate immunity, highlighting its potential as a target for the development of anti-alphaherpesvirus therapeutics.

Interferons activate JAK-STAT signaling to induce the expression of hundreds of interferon-stimulated genes to trigger the host antiviral response (23). ISGs exert antiviral effects through diverse mechanisms, targeting nearly every stage of the viral life cycle, from entry and replication to assembly and immune modulation (24). In addition to their broad-spectrum antiviral functions, certain ISGs exert highly virus-specific inhibitory effects by directly targeting viral proteins, exploiting virus-specific entry or replication pathways (25–27). Mx proteins are ISGs with intrinsic antiviral activity. MxB, the second human Mx protein, is a potent human herpesvirus restriction factor that blocks the uncoating of viral DNA from the incoming viral capsid (28). Furthermore, MxB restricts HIV-1 by direct binding to the HIV-1 core, thereby preventing the uncoating process of HIV-1 (29). TRIM5 is a RING domain E3 ubiquitin ligase with potent antiretroviral function by recognizing the multimerized capsid proteins and blocking reverse transcription (30). TRIM5 also restricts replication of specific flaviviruses such as tick-borne encephalitis virus (TBEV) serogroup, but not YFV, DENV, and ZIKV, by targeting viral proteases for proteasomal degradation (31). ISGs that inhibit PRV, such as IFITM2, have also been identified in our screening. They are reported to exert an early antiviral function in PRV infection (32). Likely, Nectin4 also shows potent antiviral activity against broad-spectrum viruses through blocking viral-cellular membrane fusion (33). However, to date, the virus-specific mechanism by which Nectin4 inhibits viral infection has not been identified.

Alphaherpesvirus employs multiple glycoproteins for attachment, receptor interaction, and membrane fusion. The initial attachment is primarily mediated through interactions of viral glycoproteins gC and/or gB with cell-surface heparan sulfate proteoglycans (2), whereas productive entry requires engagement of gD with functional entry receptors such as Nectin1. Receptor binding by gD initiates the fusion cascade through activation of downstream viral glycoproteins, including gH/gL and gB, ultimately leading to membrane fusion and viral internalization (34). In HSV-1, binding of Nectin1 to gD induces conformational rearrangements required for activation of the fusion machinery (35). In this study, we report a virus-specific antiviral mechanism of Nectin4 during PRV infection. PRV gD can interact with Nectin4, but this interaction is nonproductive for viral entry. Instead, Nectin4 may function as a decoy or nonfunctional receptor that sequesters gD away from functional entry receptors, thereby impairing the initiation of the downstream fusion process required for viral internalization. These observations support the concept that receptor binding alone is insufficient for productive alphaherpesvirus entry and that successful infection requires engagement of specific receptors capable of activating the viral fusion cascade. Although we did not directly examine whether Nectin4 affects the interactions between gD and downstream viral glycoproteins such as gH/gL, it is possible that Nectin4 binding stabilizes gD in a conformation incompatible with productive activation of the fusion machinery. In addition, our findings suggest that competitive interference with gD-Nectin1 interaction is sufficient to impair productive PRV entry. Although Nectin1 is the predominant entry receptor for PRV (36), alternative receptors such as vimentin (37) and NRP1 (38) have also been identified. Future studies will also be required to determine whether the inhibitory effect of Nectin4 on gD-Nectin1 interaction is also applicable to the gD-vimentin and gD-NRP1 interactions. Furthermore, the antiviral role of interferon-induced Nectin4 expression is likely more relevant to restricting viral spread than preventing primary infection. PRV entry into susceptible cells occurs rapidly after viral attachment, making it unlikely that interferon-mediated upregulation of Nectin4 would effectively block infection of the initially exposed cell. Instead, elevated Nectin4 expression may function during subsequent rounds of infection by competitively interfering with gD interaction with functional receptors on neighboring cells, thereby limiting viral dissemination.

Even though Nectin4 is an interferon-stimulated gene, it has been identified as an epithelial receptor for morbilliviruses, including measles virus (MV), phocine distemper virus (PDV), canine distemper virus (CDV), and peste des petits ruminants virus (PPRV) (39). This apparent discrepancy is analogous to that observed with the SARS-CoV-2 entry receptor ACE2. Ziegler et al. reported that SARS-CoV-2 receptor ACE2 is an interferon-stimulated gene in human airway epithelial cells (40). Onabajo et al. identified a novel truncated isoform of ACE2 (dACE2), but not full-length ACE2, that is induced by interferons and viruses in various human cell types (41). dACE2 lacks 356 amino-terminal amino acids and therefore cannot bind the SARS-CoV-2 spike protein. Nectin4 has two isoforms produced by alternative splicing, with isoform b lacking amino acids 412–436, which is often found in mice (42). It is intriguing to determine whether Nectin4 has multiple transcript variants, some of which are induced by interferon, whereas others are not interferon-stimulated genes but instead function as viral receptors. In addition, Nectin4 serves as the entry receptor for morbilliviruses but acts as a restriction factor for PRV. It can competitively bind PRV gD with Nectin1, thereby blocking PRV entry. Similarly, Nectin1 is the receptor for herpesviruses but functions as a restriction factor for flavivirus infection by competing with CD46 for binding to the flavivirus E2 protein (43). Thus, viral receptors can display functional plasticity, acting either as entry receptors or as restriction factors depending on the viral context.

In the battle with the host, viruses have evolved many strategies to counteract the host immune system. PRV encodes various proteins to overcome the interferon-induced antiviral state. UL41 and UL13 negatively regulate type I IFN antiviral activities by targeting IRF3 and phosphorylation (44). EP0 antagonizes the type I interferon response via inhibiting IRF9 transcription (45). PRV gE can inhibit cGAS-/STING-mediated IFN-β production by degrading CBP to interrupt the enhanced assembly of IRF3 and CBP (46). In our study, Nectin4, induced by interferon, suppresses PRV entry by directly interacting with the PRV gD protein, not through feedback regulation of the IFN signaling pathway. Besides PRV gD, the gK protein was also found to interact with Nectin4. However, interaction with gK is not required for the antiviral activity of Nectin4. Whether gK affects the biological function of Nectin4 through this interaction remains to be explored in the future. Furthermore, PRV encodes numerous proteins. Thus, in addition to targeting the upstream and downstream signaling of IFN, the viral proteins that directly interact with and modulate Nectin4 are required to be identified.

## MATERIALS AND METHODS

### Cells and viruses

A549, HEK293T, Vero, and Huh7 cells were obtained from American Type Culture Collection and cultured in high-glucose Dulbecco’s modified Eagle’s medium (DMEM, Gibco) supplemented with 10% fetal bovine serum (FBS, Gibco) and 1% penicillin/streptomycin (Gibco). HeLa cells were obtained from the American Type Culture Collection and cultured in RPMI 1640 (Gibco) supplemented with 10% fetal bovine serum and 1% penicillin/streptomycin. Immortalized porcine alveolar macrophages (3D4/21) and Chinese hamster ovary (CHO) cells were purchased from Fuheng Biology (Shanghai, China) and cultured in RPMI 1640 supplemented with 10% FBS and 1% penicillin/streptomycin. CaoV3 cells (ovarian cancer cells) were obtained from Fuheng Biology (Shanghai, China) and cultured in DMEM supplemented with 10% FBS and 1% penicillin/streptomycin. Immortalized bone marrow-derived macrophages (iBMDMs) were kindly provided by Professor Xin Lin (Tsinghua University, Beijing, China). The PRV and PRV-GFP were stored in our laboratory. The PRV-ΔgK generated by replacing the PRV UL53 gene with GFP was provided by Professor Zongyi Bo (Yangzhou University, Yangzhou, China). HSV-1 was kindly provided by Professor Yingjie Sun (Shanghai Veterinary Research Institute).

### Reagents and antibodies

Puromycin was purchased from Beyotime (Shanghai, China). Poly(dA:dT) and Poly(I:C) were obtained from Invivogen (Beijing, China). Anti-DYKDDDDK(Flag) and anti-Myc magnetic beads were from MedChemExpress (HY-K0207 and HY-K0206, China). Did perchlorate was purchased from MedChemExpress (HY-D1028, China). The following antibodies were used: DYKDDDDK tag monoclonal antibody (1:3,000, M20008L, Abmart, China), V5 tag polyclonal antibody (1:2,000, 14440-1-AP, Proteintech, China), MYC tag polyclonal antibody (1:2,000, R20002S, Abmart, China), anti-Nectin4 polyclonal antibody (1:1,000, 21903-1-AP, Proteintech, China), GAPDH monoclonal antibody (1:10,000, TA802519M, Origene), and goat HRP-anti-mouse/rabbit IgG (H + L) secondary antibody (1:5,000, BF03001/BF03008, Biodragon). Anti-HSV-ICP0 monoclonal antibody (1:1,000, sc-56985, Santa Cruz) and anti-PRV gE/anti-PRV UL42 were kindly provided by Professor Yiping Wang (Sichuan Agricultural University, Sichuan, China). Anti-PRV gB monoclonal antibody was kindly provided by Professor Changchao Huan (Yangzhou University, Yangzhou, China). Recombinant Human Nectin-4 Protein (ECD, His Tag) was purchased from Sinobiological (19771-H08H). The PRV-gD protein was purchased from Antibody System (EVV32901). The purified human Nectin1 protein was purchased from MedChemExpress (HY-P70494).

### Plasmid, siRNA, and transfection

The Nectin4 gene of humans and pigs was synthesized by Genewiz (Suzhou, China) and then subcloned into pHAGE-MCS-3×Myc and pECMV-3 ×FLAG, respectively. The human Nectin1 cDNA, amplified from A549 cells, was subcloned into pHAGE-MCS-Puro-3×Myc and pECMV-3 ×FLAG. PRV genes encoding glycoprotein were synthesized on the backbone of pHAGE-MCS-Puro-3×Myc by Genewiz (Suzhou, China). The Nectin4-dEC/dTM/dIC/dIgV/dIgC1/dIgC2 truncations were amplified and inserted into pECMV-3 ×FLAG. For transient expression, JetPrime transfection reagent (PolyPlus, France) was used to introduce the plasmids into the cells. Briefly, the cells were transfected with the indicated plasmids and then changed to fresh medium at 8 h post-transfection. After 24 h, cells were collected for further analysis. siRNA-mediated knockdown assays were executed by Lipofectamine RNAiMAX (Thermo Fisher, USA). The knockdown efficiency was tested at 48 h after transfection. Empty vectors or siRNA that did not target any genes (siNC) were used as negative controls for transfection experiments. Full siRNA sequence information is provided in Table S2.

### Generation of stable cell lines expressing Nectin4

Stable cell lines (A549, Vero, and CHO) expressing Nectin4 were generated using the lentiviral system. Either an empty lentivirus plasmid or a Nectin4 lentivirus plasmid was transfected with pMD2.G and psPAX2 into HEK293T cells using PEI. Supernatants were harvested at 48 h after transfection and then incubated with the indicated cells for 72 h. Positive cells were selected with puromycin and verified by Western blotting assay. Cell viability was determined by the CCK-8 assay. Briefly, cells were seeded into 96-well plates at a density of 1 × 10³ cells per well and cultured for 12 or 24 h. At each time point, 10 μL of CCK-8 solution was added to each well, followed by incubation for 1 h at 37°C. The absorbance at 450 nm was then measured using a microplate reader, and the average relative absorbance was calculated.

### qPCR

Total cellular RNA was extracted by RNAiso Plus (SD1412, TaKaRa) and reverse-transcribed into cDNA using HiScript II Q RT SuperMix for qPCR (Vazyme). qPCR was performed using the ChamQ Universal SYBR qPCR Master Mix (Vazyme) on an Applied Biosystems QuantStudio 5 (Thermo Fisher Scientific). Relative quantification of gene expression was normalized using GAPDH as a control. Viral genome copy number quantification based on UL27 was performed using the intracellular or supernatant viral genome as a template. RT-qPCR was performed with the following cycling conditions: 3 min at 95°C, 40 cycles of 5 s at 95°C, 15 s at 60°C, and 20 s at 72°C. Data were analyzed with the 2^−ΔΔCT^ method. All quantitative experiments were set up with at least two replicates and tested in triplicate to avoid test and instrument errors. Primers used for qPCR are in Table S1.

### ISG screen

The ISG library, which consists of 268 ISGs stimulated by type I and type II interferons, was kindly provided by Prof. Genhong Cheng (University of California, Los Angeles). For the first-round screen, HEK293T cells were seeded in 48-well plates at a density of 2 × 10^4^/well and transfected with 268 ISGs plasmids for 24 h. The cells were then challenged with PRV-GFP (MOI of 0.1) for 24 h, and the viral genome in the cell supernatant was extracted for qPCR. Sixteen ISGs with potent antiviral activity from the first-round screen were further validated. Briefly, HEK293 cells were transfected with 16 ISGs individually and then challenged with PRV for 24 h. The cell supernatant was collected for the TCID_50_ assay.

### Immunoprecipitation and Western blot

Cells were lysed with IP buffer (50 mM Tris-Cl pH 8.0, 5 mM EDTA, 150 mM NaCl, and 0.5% NP-40) plus protease inhibitor cocktail (C0001, TargetMol, China). Whole-cell lysates were obtained by centrifugation at 12,000 rpm for 15 min at 4°C and then incubated with anti-FLAG or anti-MYC magnetic beads at 4°C for 3 h. The immune complexes were then washed with cold RIPA buffer four times and then separated by 10% SDS-PAGE for immunoblot analysis with the indicated antibodies.

Soluble protein extraction was performed with RIPA buffer (50 mM Tris-HCl [pH 7.5], 150 mM NaCl, 0.5% sodium deoxycholate, and 1% NP-40) supplemented with the protease inhibitor (C0001, TargetMol, China). The cells were lysed by shaking at 4°C for 15 min and centrifuged at 12,000 × *g* for 10 min to collect the supernatant. Whole protein extraction was performed with sodium dodecyl sulfate (SDS) buffer (25 mM Tris-HCl [pH 7.5], 150 mM NaCl, and 2% SDS). Samples were heat-denatured at 95°C for 10 min. Equivalent protein aliquots were subjected to SDS-PAGE and transferred to the nitrocellulose membrane. After incubation with primary antibodies for 12 h at 4°C and secondary antibodies for 2 h at room temperature, chemiluminescence was visualized using the Tanon 5200 Chemiluminescent Imaging System.

### Virus preparation and viral titer determination

PRV stock was prepared using Vero cells. Debris was removed by centrifugation at 8,000 × *g* for 30 min, and viral particles were collected by centrifugation at 20,000 × *g* for 2 h. The virus particles were resuspended in PBS and then centrifuged over a sucrose density gradient (20%–50%) at 20,000 × *g* for 3 h at 4°C. The virus particles were resuspended in PBS and stored at −80°C.

The virus titer was measured by the classical TCID_50_ assay. Briefly, Vero cells were seeded into 96-well plates at 1 × 10^3^/well for 12 h, and then the virus samples were prepared with a series of 10-fold dilutions. After 72 h, a cytopathic effect was observed, and TCID_50_ was calculated.

### Virus attachment and internalization assay

For the attachment assay, cells were seeded in 12-well plates at a density of 1 × 10^5^/well and transfected with plasmids for 24 h. Cells were placed on ice and precooled for 30 min, followed by the virus (MOI of 1) incubation at 4°C for 2 h, after which the cells were washed three times with cold PBS to remove the unbound virus. The attached virus was determined based on viral genome copy number using qPCR.

For the virus internalization assay, cells were placed on ice and precooled for 30 min, followed by the virus (MOI of 1) incubation at 4°C for 2 h, after which cells were washed three times with cold PBS to remove the unbound virus. After an additional 1 h incubation at 37°C, cells were washed with acid citrate buffer (40 mM sodium citrate, 10 mM KCl, and 135 mM NaCl, at pH = 3.0) (300 µL/well) three times to remove any viruses that do not enter the cells. Viral genomes were collected for the viral genome copy number quantification by qPCR.

### Virus particle labeling

Purified PRV was labeled by incubation with 200 nM Did in PBS for 2 h at room temperature in the dark. The labeled virus was then concentrated using an Amicon Ultra centrifugal filter (UFC9100, Merck) at 4,000 × *g* for 1 h, washed twice with PBS, and resuspended to obtain Did-PRV. To assess viral attachment, the Vero cells expressing Nectin4 were infected with Did-PRV and incubated at 4°C for 1 h, followed by PBS washes to remove the unbound virus. To assess viral internalization, cells were shifted to 37°C for 1 h in complete medium and then washed three times with acidic buffer solution. Viral attachment and internalization were visualized by a Zeiss LSM980 confocal microscope (63× oil immersion objective).

### Statistical analysis

All data are presented as mean ± SD. Statistically significant differences were determined using a two-tailed independent Student’s *t*-test. Statistical significance was defined as *P < 0.05, **P < 0.01, and ***P < 0.001 versus controls.

## Data Availability

The data that support the findings of this study are available from the corresponding author upon reasonable request.

## SUPPLEMENTAL MATERIAL

Supplemental material jvi.00788-26-s0001.docx Fig. S1 to S4; Tables S1 and S2.

ASM does not own the copyrights to Supplemental Material that may be linked to, or accessed through, an article. The authors have granted ASM a non-exclusive, world-wide license to publish the Supplemental Material files. Please contact the corresponding author directly for reuse.
