# Supplementary material for: Nectin4 restricts pseudorabies virus infection by blocking gD-Nectin1 interaction
Source: J Virol. 2026 Jul 20;100(8):e00788-26. doi: 10.1128/jvi.00788-26 (PMC13483241; doi:10.1128/jvi.00788-26)
Supplement: Supplementary file 1 [file jvi.00788-26-s0001.docx]

**SUPPLEMENTARY INFORMATION**

**Supplementary Figures 1-4**

**Table S1 and S2**

**FigS1**. **Restriction factor Nectin4 is an ISG.**

Nectin4 is activated by interferon in various cells. (A) iBMDM cells were treated with type I interferon (500 ng/mL) for 12h, transfected with DNA analogues (Poly(da:dt), 500 ng/well) or RNA analogues (Poly (I: C),500 ng/well) for 6 h, and the transcriptional level of Nectin4 mRNA was detected by qPCR. (B) PAM cells were treated with type I interferon (500 ng/mL) for 12h, and the transcriptional activation level of Nectin4 mRNA was detected by qPCR. (C) iBMDM cells were treated with type I interferon (500 ng/mL) for 12h. The Nectin4 was detected by Western Blot. (D) PAM cells were treated with type I interferon (500 ng/mL) for 12h. The Nectin4 was detected by Western Blot. (E)The endogenous protein expression levels of Nectin4 were detected in HEK293, HeLa, A549, Huh7, and Caov3 cells by Western Blot. (F) ISGs were transfected into HEK293T cells for 48 hours, and cell activity was detected by CCK8 assay. ***P < 0.001 by unpaired t-test. Graphs show the mean ± SD.

**FigS2**. **Nectin4 inhibits herpesvirus replication.**

HeLa cells and A549 cells were transfected with the Nectin4 plasmid for 24 h and infected with PRV (MOI of 0.1) for 24 h. The transcription level of PRV-gB was detected by qPCR **(A, B)**. The empty plasmid was used as a negative control. HeLa cells were transfected with the Nectin4 expression plasmid for 24 h and infected with HSV-1 (MOI of 1) for 24 h. The viral protein and Nectin4 were detected by Western Blot **(C)**. The transcription level of HSV-gB was detected by qPCR **(D)**. A549 cells were transfected with the human and porcine Nectin4 plasmid for 24 h and infected with PRV (MOI of 0.1) for 24 h. GFP fluorescence was observed **(E)**, and the transcription level of PRV-gB was detected by qPCR(F). **(G, H)** HeLa and A549 cells were treated with different concentrations of the JAK1 inhibitor Filgotinib for 12 or 24 hours, and cytotoxicity was determined using a CCK8 assay kit. The inhibitory activity of Filgotinib on interferon signaling was tested. Poly(dA:dT) and Poly (I: C) were transfected into HeLa **(I)** and A549 **(J)** cells for 6 h, with or without Filgotinib. The ISG20 mRNA transcription level was detected by qPCR. (K) HeLa cells were transfected with Flag-EV or Nectin4 plasmid for 24 h and treated with 1U/mL heparanase for 3 h. Then, cells were infected with PRV (MOI of 1) for attachment assays, and viral genome copy number was determined by qPCR. HeLa cells transfected with the Nectin4 plasmid were infected with HSV-1 (MOI of 1) for attachment (L) and internalization assays (M). *P < 0.05, **P < 0.01, and ***P < 0.001 by unpaired t-test. Graphs show the mean ± SD.

**FigS3. Nectin4 exhibits antiviral activity through its IgV-like domain.**

**(A, B)** Full-length Nectin4 and its different mutants were transfected into HEK293T for 24 h, followed by PRV-GFP (MOI of 0.1) infection for 24 h. GFP fluorescence imaging was captured, and UL42 protein was detected by Western Blot in HEK293T cells. HeLa **(C)** and A549 **(D)** cells were transfected with full-length Nectin4 and IgV-like deletion mutant for 24 h, then infected with HSV-1(MOI of 1) for 24 h. The viral protein ICP0 was detected by WB, and the transcription level was detected by qPCR. *P < 0.05, **P < 0.01, and ***P < 0.001 by unpaired t-test. Graphs show the mean ± SD.

**FigS4. Nectin4 interacted with gK but did not affect its antiviral activity.**

**(A)** PRV-wt and PRV-ΔgK were infected into 293T cells. At different times, the supernatants and cells were collected, respectively, for viral titer determination by TCID_50_ assay. **(B)** HeLa cells were transfected with the full-length or IgV domain-deficient Nectin4. After 24 hours of PRV-ΔgK infection (MOI of 0.1), the GFP fluorescence of the infected cells was observed. HeLa cells were transfected with Nectin4-FL or Nectin4-dIgV for 24 h, then infected with either PRV-wt or PRV-dgK (MOI of 1) for 8 h. The transcription level of PRV-gB (C), and the expression of PRV-gE and UL42 were detected by RT-qPCR and Western Blot **(D)**. *P < 0.05 by unpaired t-test. Graphs show the mean ± SD.

**Supplementary Table 1. The primers used in this study**

| Hum-GAPDH-F | ACCCAGAAGACTGTGGATGG |
| --- | --- |
| Hum-GAPDH-R | TCAGCTCAGGGATGACCTTG |
| Mus-GAPDH-F | AACGGATTTGGCCGTATTGG |
| Mus-GAPDH-R | CATTCTCGGCCTTGACTGTG |
| Monkey-GAPDH-F | TCGTGGAAGGACTCATGACC |
| Monkey-GAPDH-R | CCATCCACAGTCTTCTGGGT |
| Pig-GAPDH-F | TGGAAAGGCCATCACCATCT |
| Pig-GAPDH-R | ATGGTCGTGAAGACACCAGT |
| Hum-PVRL4-F | GACCACGGTGAGGGAGATAG |
| Hum-PVRL4-R | GGGTCCCATTCTCCTGAACA |
| Pig-PVRL4-F | TCCGCTGTCACTTCAGAGTT |
| Pig-PVRL4-R | AGCTCCTTCTCTGCCAACTT |
| Mouse-PVRL4-F | AGGAGACACTCTGGGCTTTC |
| Mouse-PVRL4-R | AAGCTCATTGCTGACGTGAC |
| PRV-UL27-F | AAGTTCAAGGCCCACATCTA |
| PRV-UL27-R | TGAAGCGGTTCGTGATGG |
| HSV-UL27-F | AACGCGACGCACATCAAG |
| HSV-UL27-R | CTGGTACGCGATCAGAAAGC |
| Monkey-IFNb-F | TCCTGTTGTGCTTCTCCACT |
| Monkey-IFNb-R | TGCGGCTGCTTAATTTCCTC |
| Hum-ISG20-F | GAGATCACCGATTACAGAACC |
| Hum-ISG20-F | GTGTCGTAGATTGTGTAGCC |

**Supplementary Table 2. siRNA oligos used in this study**

| siPVRL4-1 sense (5'-3') | CUGAUAGUCUCAAGGACAAdTdT |
| --- | --- |
| siPVRL4-1 antisense (5'-3') | UUGUCCUUGAGACUAUCAGdTdT |
| siPVRL4-2 sense (5'-3') | GUCACCUCAGAGUUCCACUdTdT |
| siPVRL4-2 antisense (5'-3') | AGUGGAACUCUGAGGUGACdTdT |
| siPVRL4-3 sense (5'-3') | ACUCCAAAUACGGGCUUCAdTdT |
| siPVRL4-3 antisense (5'-3') | UGAAGCCCGUAUUUGGAGUdTdT |
